# The Patriotism of the Nurse-Training Schools

**Published:** 1917-04-14

**Authors:** 


### THE PATRIOTISM OF THE NURSE-TRAINING SCHOOLS.

### XIII. Some Lancashire Institutions.

Lancashire, which can boast of many historic foundations for the sick, and has been the pioneer in municipal training schools for nurses, is among the foremost counties in sending nurses out to take duty under the naval authorities. Some part of the nurses' endeavours for the wounded in this country has already been published in these columns, but the tale is not yet complete.

### Royal Infirmary, Manchester.

The staff in this hospital numbers 190, and the following lists show the extent to which it was depleted during the first few months of war, the vacancies being, of course, filled up by other nurses trained in this well-known school for nurses.

*Q.A.I.M.N.S.R.*—Misses Irwin, Sitton, Lumsden, Croft, J. Coulter, Knox, Baird, McDiarmid, Fernel, Drummond. *T.F.N.S.*—Misses Hills, Simpson, James, Dawson, Davidson, Goodrick, Gowie, Goodacre, K. Scott, Gooseman, Sloan, Shaw, Robinson, Cannell, Jackson. Thirty-two nurses holding the Manchester Infirmary certificate went from other institutions to join Q.A.I.M.N.S.R., and twenty to join the T.F.N.S. The Misses Scorgie, Skea, and Peters went to Serbia with the Scottish Women's Hospital. During the past year the following have received the decoration of the Royal Red Cross (Second Class):—Miss A. Hills, Miss E. C. Sutton, Miss E. Sloan, Miss M. Cowie, Miss E. Kay, Miss J. Fairgrieve, and Miss J. Prentice.

The following have been mentioned in dispatches:—Miss K. Davidson, Miss M. James, Miss E. Sloan, Miss A. Hills, Miss M. Cowie (twice), Miss A. Lofthouse, Miss M. S. Lumsden.

### Ancoats Hospital, Manchester.

On war being declared five sisters out of a total of nine were called up to join the Territorial unit to which they were attached, their posts being filled temporarily by staff nurses. Since then fourteen other members of the nursing staff have left for military work. Sister Bell has gone to Egypt, Sister Armstrong to France, Sister Jones, late matron of the Ancoats Convalescent Home, is now matron of the 2nd Western General Hospital, Reddish, Manchester. The following sisters and nurses have been appointed to military hospitals at home:—Theatre Sister Pickett, Sister Mellowdew, Staff-Nurse Aston, Sister Sykes, Sister Cooper, Nurses Burns, Wolverton, and Smethurst. Nurse Perks has gone to Malta.

### Liverpool Stanley Hospital.

A number of the nursing staff were members of the T.F.N.S. at the outbreak of war, and others promptly "joined up" as they became eligible.

With two exceptions, they are all on foreign service. Their names are:—Sister Demain, Nurses Frampton, Mitchell, Clubb, Burbridge, Green, Thompson, Curtis, and Iddon.

Sisters Hay and Bowman are on home service.

### David Lewis Northern Hospital, Liverpool.

The following is a list of those called up from this hospital, and who are still engaged in military or naval nursing abroad or at home:—Sisters Ryder, Vallance, Spence, Anderson, Roberts, Clark, Coates, and Richardson; Nurses Williams, Gaw, Bach, Hollows, Wright, Cain, Stewart, Folkhard, Ryland, B. Davies, Bomphrey, Griffiths, Cowperthwaite, A. M. Atkinson, L. Davies, Hyde, Preston, Lloyd, N. Roberts, and Belton.

